# Extracorporeal Shock Wave Therapy Provides Limited Therapeutic Effects on Carpal Tunnel Syndrome: A Systematic Review and Meta-Analysis

**DOI:** 10.3390/medicina58050677

**Published:** 2022-05-19

**Authors:** Ko-Ta Chen, Yu-Pin Chen, Yi-Jie Kuo, Ming-Hsiu Chiang

**Affiliations:** 1 Department of Orthopedics, Taipei Medical University Hospital, Taipei 110301, Taiwan; kotahero@gmail.com; 2 Department of Orthopedics, School of Medicine, College of Medicine, Taipei Medical University, Taipei 11031, Taiwan; 99231@w.tmu.edu.tw (Y.-P.C.); benkuo5@tmu.edu.tw (Y.-J.K.); 3 Department of Orthopedics, Wan Fang Hospital, Taipei Medical University, Taipei 11696, Taiwan; 4 Department of General Medicine, Kaohsiung Chang Gung Memorial Hospital, Kaohsiung 83301, Taiwan

**Keywords:** carpal tunnel syndrome, extracorporeal shock wave therapy, night wrist splint, pain, recovery of function, meta-analysis

## Abstract

Night wrist splinting has been a conservative treatment for carpal tunnel syndrome. The addition of extracorporeal shock wave therapy provides an alternative treatment. However, strong evidence on the clinical effectiveness of extracorporeal shock wave therapy for carpal tunnel syndrome is still lacking. This study aimed to investigate the effectiveness and safety of extracorporeal shock wave therapy compared with treatments of night wrist splints alone for patients with carpal tunnel syndrome. In this systematic review and meta-analysis, no limitation criteria were used for study selection. All available articles that compare the effectiveness between extracorporeal shock wave therapy combined with night wrist splint and night wrist splint alone for treating carpal tunnel syndrome published up to 20 January 2022 were identified from the databases of PubMed, Embase, and Cochrane Central Register of Controlled Trials Central. The primary outcomes were a standard mean difference with a 95% confidence interval on the improvement of symptom severity and functional impairment between the two groups. In an attempt to analyze trends over time in studies that report repeated measurements, an all time-points meta-analysis (ATM) was undertaken. Seven randomized controlled trials with a total of 376 participants were included in this study. Significant improvements in functional impairment and symptom remission were only observed in the extracorporeal shock wave group at four weeks post-treatment. Extracorporeal shock wave therapy did not demonstrate superior efficacy compared to treatment with night wrist splint alone at 8–10 and 12–14 weeks post-treatment, or through the ATM approach. In conclusion, the therapeutic effect of extracorporeal shock wave therapy is transient and mostly nonsignificant compared with using night wrist splint alone. No serious side effects were reported in all included studies. Other conservative treatments to ameliorate carpal tunnel syndrome symptoms are warranted.

## 1. Introduction

Various treatments are available for the management of carpal tunnel syndrome (CTS). Although surgical release of the flexor retinaculum for decompression of median nerve within carpal tunnel is the definite treatment for severe CTS with medium- and long-term effectiveness [1], concerns still exist on the associated surgical complications, with pillar pain reported by up to 38% after surgical decompression of CTS. Conservative treatments, which are regarded as the first-line treatment for CTS, include manual physical therapy, wrist splints, and corticosteroid usage [2]. However, long-term results for these conservative treatments remain poor or uncertain, leaving space for more effective conservative treatments prior to surgical release for CTS [3].

Extracorporeal shock wave therapy (ESWT) is a relatively new method that has been used to treat a variety of musculoskeletal diseases. In addition to direct mechanical effects, ESWT also acts at the biological level by interacting with various tissues and cell elements by through stimulations [4]. In an animal study, ESWT demonstrated anti-inflammation activity by increasing endogenous nitric oxide production and downregulating NF-kappa B activation [5]. Preliminary case reports show that ESWT greatly improves CTS patients’ symptoms and function [6]. One previous meta-analysis also reported that ESWT had promising treatment benefits on CTS patients. Nevertheless, the study included discrepant control groups, which may lead to greatly biased conclusions [7]. Evidence with a more prudent approach is still required to justify the role of ESWT in the treatment of CTS. This meta-analysis strictly selected all eligible studies in order to provide clinical evidence on the effectiveness and safety of ESWT compared with treatments of night wrist splints alone for patients with CTS.

## 2. Materials and Methods

The meta-analysis was performed in a manner consistent with the Preferred Reporting Items for Systematic Reviews and Meta-Analyses (PRISMA) statement [8].

### 2.1. Inclusion and Exclusion Criteria

Randomized controlled trials (RCTs) or prospective cohort studies that evaluated the outcomes of patients with CTS who were treated with ESWT with wrist splint and those treated with wrist splint alone were reviewed. Furthermore, reviewed studies had to clearly report inclusion and exclusion criteria for patients, treatment protocols, the ESWT frequency, and definitions and values of outcome parameters. Studies that enrolled patients aged <18 years, duplicated patient cohorts, or which were review articles or case reports were excluded. 

### 2.2. Search Strategy and Identification of Eligible Papers

Two authors searched PubMed, Embase, Cochrane Library using the keywords ‘“extracorporeal shock wave therapy” OR “shock wave” OR “ESWT”’ AND ‘“carpal tunnel syndrome” OR “Median Neuropathy” OR “Compression Neuropathy” OR “Entrapment Neuropathy”’ AND ““splint” OR “conservative treatment”” from the inception of the earliest records to 20 January 2022 without any limitations. The reference lists of review articles relevant to this topic were manually searched to identify potentially eligible papers.

After the searches had been completed, duplicate works were removed and two authors independently screened titles and abstracts. Both authors then applied the eligibility criteria through reading the full texts and developed a final list of studies to be included. Any disagreements were resolved by a third reviewer.

### 2.3. Appraisal of Methodological Quality

Two authors independently assessed the methodological quality of each reviewed study by using the Risk of Bias tools 2 to assess the risk of bias in randomized trials [9]. RCTs were awarded an overall risk of bias grade of high, some concerns, or low. This grade was calculated by making assessments in the following five domains: bias that arises from the randomization process, bias owing to deviation from the intended intervention, bias owing to missing outcome data, bias in the measurement of the outcome, and bias in the selection of reported results.

### 2.4. Outcome

The primary outcomes were the mean difference (MD) of the symptom severity and functional impairment between the ESWT group and the night wrist alone group at the baseline and at each follow-up. Symptom severity and function impairment of CTS were assessed by the Boston Carpal Tunnel Syndrome Questionnaire (BCTQ), which is a major tool to assess CTS. The BCTQ comprises of eight and eleven questions, respectively, for subjective functional impairment (Boston Carpal Tunnel Syndrome Questionnaire functional subscale, BCTQf) and symptoms severity (Boston Carpal Tunnel Syndrome Questionnaire symptom subscale, BCTQs) assessment, in which the patient can score from one to five points for each question. High points in these two subscales indicates serious symptoms or severe daily function impairment [10]. Secondary outcomes included a visual analog scale (VAS) for pain evaluation and electrophysiological findings between groups at baseline and at each follow-up. In an attempt to analyze trends over time in studies that reported repeated measurements, such as BCTQf, BCTQs, and VAS, an all time-points meta-analysis (ATM) was undertaken.

### 2.5. Data Extraction

Two authors independently extracted baseline and outcome data from the datasets that were provided in the reviewed studies. The study designs, study population characteristics, inclusion and exclusion criteria, ESWT frequency, adverse events, and outcome parameters were also extracted. Decisions made individually by the reviewers were compared and disagreements were resolved by a third reviewer.

### 2.6. Statistical Analyses

Included studies were categorized by the different interventions that the patient received, including ESWT plus night wrist splint or night wrist splint alone. Separate meta-analyses were performed using statistical software R [11] and the add-on package Metafor [12]. Under the presumed heterogeneity of the sample populations across all of the recruited studies, the analytical models were random-effects meta-analysis models rather than fixed-effect models [13]. All time-points meta-analysis (ATM) was conducted to analyze the trends or changes over time in repeated measurement studies; the pooled estimate at a time-point was quantitatively compared with estimates at other time-points [14]. All effect sizes on identical scales were merged using MD ± standard deviation (SD) and 95% confidence intervals (95% CIs). A two-tailed *p* value of under 0.05 was considered to indicate statistical significance.

To evaluate the statistical heterogeneity and inconsistency of the effects of treatments across studies, Cochrane Q tests and I^2^ statistics, respectively, were used. Statistical significance was set at *p* < 0.10 for Cochrane Q tests. Statistical heterogeneity across studies was assessed using the I^2^ test, which quantified the proportional total outcome variability across studies.

## 3. Results

### 3.1. Study Selection

**Figure 1:**
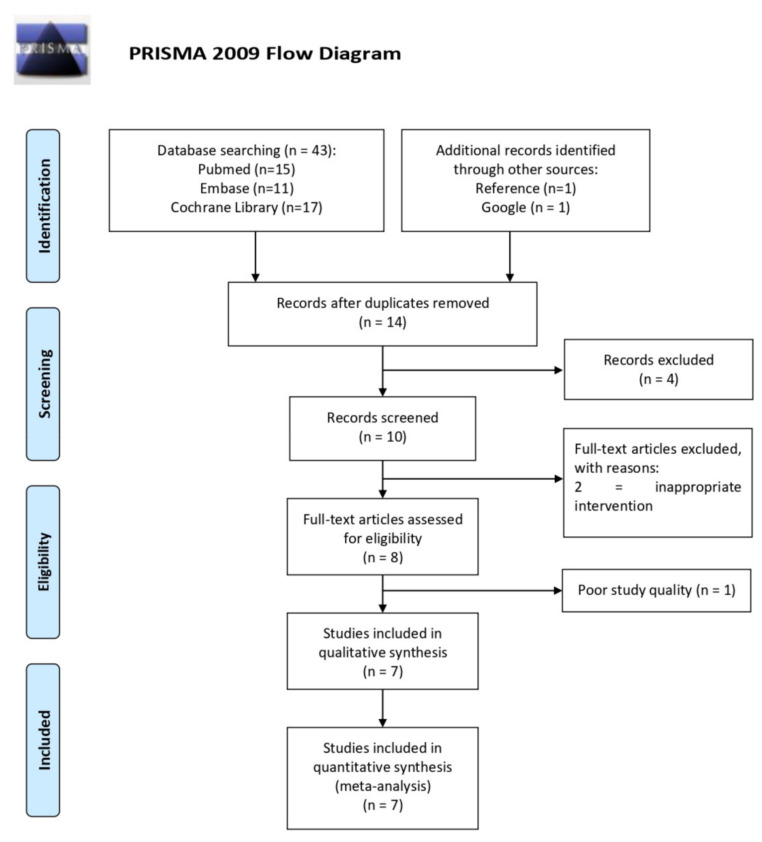
After titles/abstracts were screened, the eligibility of 10 full-text articles was assessed. Of these, seven studies were excluded, leaving seven trials for meta-analysis. The figure was accessed and produced on 8 February 2022 [8].

Following a comprehensive search, 14 studies were identified. The searching strategies for different databases are provided in Figures S1–S3 (Supplementary Materials). Screening of the titles/abstracts yielded 10 full-text articles whose eligibility was assessed. Of these, two were excluded for inappropriate intervention that did not provide wrist splint or had surgical intervention. Ultimately, the systematic review included seven articles (Figure 1) and a PRISMA checklist is provided as a Supplementary Material (PRISMA 2009 checklist) [8]. All included studies were RCTs. These seven trials were published between 2016 and 2020 and their sample sizes ranged from 20 to 97 patients. The vast majority of the baseline characteristics in each of the seven studies were balanced [15,16,17,18,19,20,21,22].

### 3.2. Methodological Quality of Included Studies

Table S1 presents the methodological quality of the included trials. Five studies had a low risk of bias, two had some concerns, and one had a high risk of bias. The research with high risk of bias was excluded [21], leaving seven studies enrolled for meta-analysis to maintain the integrity of the conclusion drawn from the present study. ESWT treatment protocols varied between studies and are presented in a descriptive manner in Table 1. The vast majority of the baseline characteristics in each of the seven studies were balanced.

### 3.3. Boston Carpal Tunnel Syndrome Questionnaire Functional Subscale

**Figure 2:**
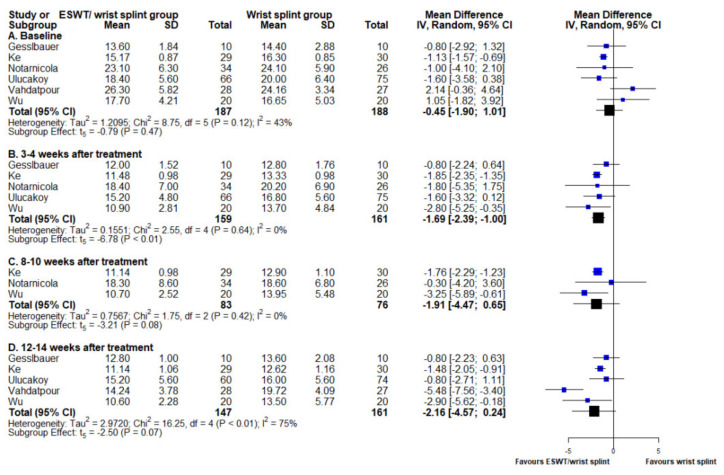
Forest plot of mean difference in BCTQf score between ESWT/night wrist splint and night wrist splint alone groups. Mean difference and standard deviation of each enrolled study and the meta-analysis results were presented as blue and black squares at the left column of the figure, respectively. BCTQf score at (A) baseline, (B) four weeks after intervention, (C) 8–10 weeks after intervention, and (D) 12–14 weeks after intervention.

Six out of seven studies provided datasets of the BCTQf at baseline and at 3–4, 8–10, and 12–14 weeks of follow-up between ESWT/night wrist splint group and night wrist splint alone group [15,16,18,19,20,22]. One study used Quick DASH [17]. Results at 3–4 week of follow-up showed a statistically significant benefit favoring the ESWT/night wrist splint group (MD −1.69, 95% CI −2.39 to −1.00, *p* < 0.001). However, the significance of improvement in functional impairment in the ESWT/night wrist splint group did not last for the following two time points (post-treatment week 8–10 and 12–14: (MD −1.91, 95% CI −4.47 to 0.65, *p* = 0.08) and (MD −2.16, 95% CI −4.57 to 0.24, *p* = 0.07), respectively). The I^2^ value at the last time point was over 50%, indicating high heterogeneity across the studies (Figure 2).

**Figure 3:**
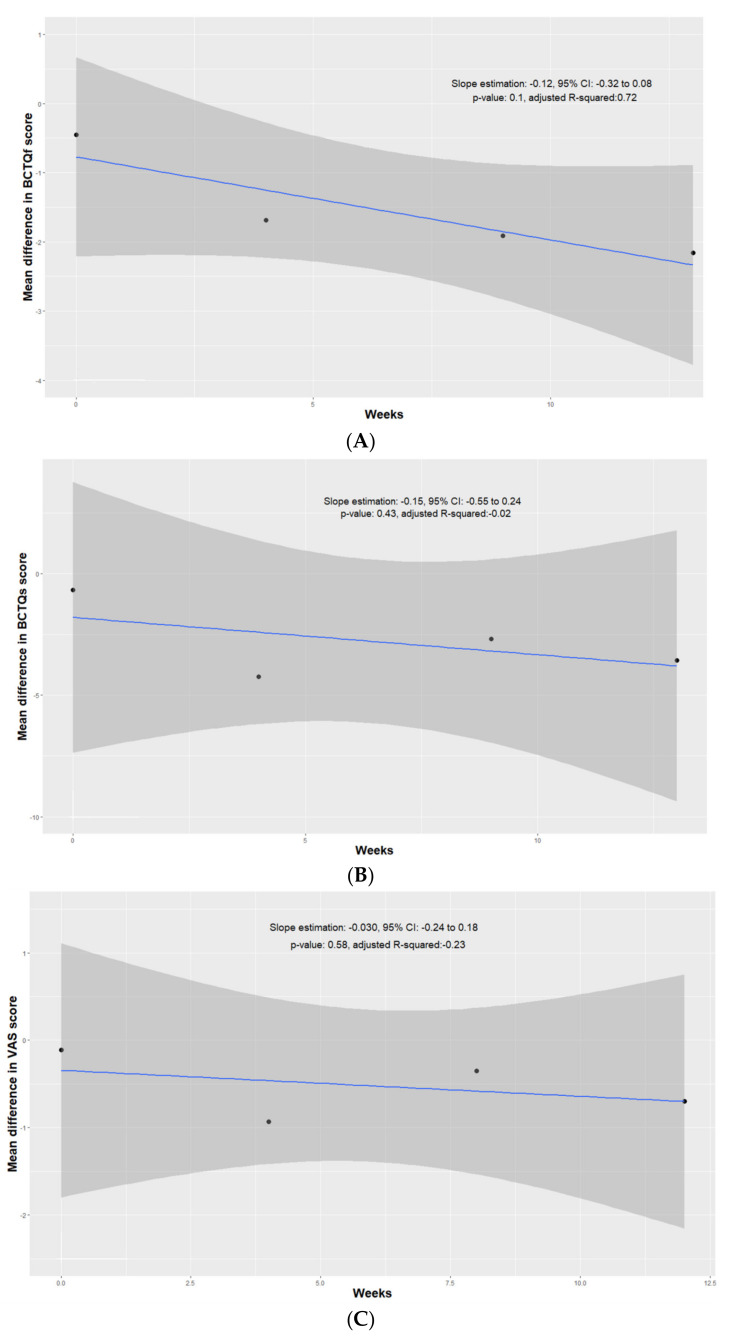
Linear regression model of (**A**) pooled BCTQf score, (**B**) pooled BCTQs score, and (**C**) pooled VAS score evaluating ESWT/night wrist splint and night wrist splint alone groups at baseline, 4 weeks, 9 weeks, and 13 weeks after treatments.

In an attempt to quantify the variation difference between the BCTQf over time following index treatment between the ESWT/night wrist splint and night wrist splint alone groups, an ATM approach was undertaken [14]. In brief, a linear regression model was established by analyzing the pooled results at baseline and 4, 9, and 13 weeks post-treatment. The x-axis represents the follow-up period and the y-axis represents the MD between the ESWT/night wrist splint and night wrist splint alone groups. The negative slope shows that the ESWT/night wrist splint arm had lower BCTQf than the night wrist splint, arm with an average decrease of 0.12 points per week (95% CI −0.32 to 0.08, *p* = 0.1), indicating that the ESWT/night wrist splint group did not have significantly more rapid improvement on BCTQf over time than the night wrist splint alone group within 3 months after index treatment (Figure 3A).

### 3.4. Boston Carpal Tunnel Syndrome Questionnaire Symptom Subscale

**Figure 4:**
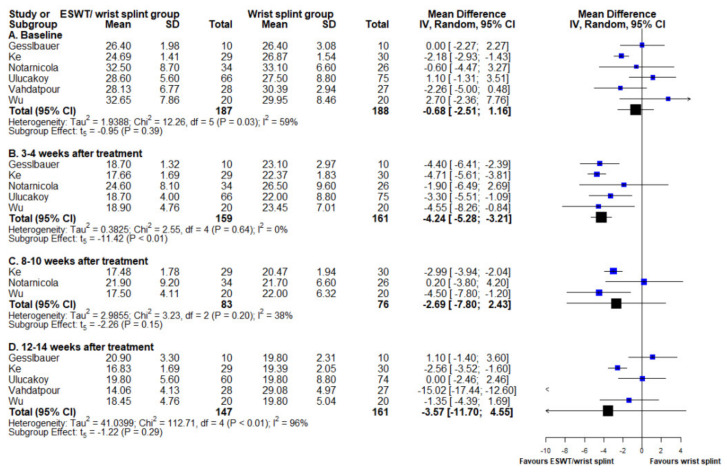
Forest plot of mean difference in BCTQs score between ESWT/night wrist splint and night wrist splint alone groups. Mean difference and standard deviation of each enrolled study and the meta-analysis results were presented as blue and black squares at the left column of the figure, respectively. BCTQs score at (A) baseline, (B) 4 weeks after intervention, (C) 8–10 weeks after intervention, and (D) 12–14 weeks after intervention.

The same six studies that reported BCTQf datasets also had BCTQs results at baseline and at 3–4, 8–10, and 12–14 weeks of follow-up [15,16,18,19,20,22]. The ESWT/night wrist splint group had non-significantly lower symptom severity at baseline (MD −0.68, 95% CI −2.51 to 1.16, *p* = 0.39). At post-treatment 3–4 weeks, the ESWT/night wrist splint group demonstrated significant lower symptoms severity compared to that of the night wrist splint group (MD −4.24, 95% CI −5.28 to −3.21, *p* < 0.001). Similar to the findings of the BCTQf, the significance of improvement on symptom severity in the ESWT/night wrist splint group did not last over the following two time points (post-treatment week 8–10 and 12–14: (MD −2.69, 95% CI −7.80 to 2.43, *p* = 0.15) and (MD −3.57, 95% CI −11.70 to 4.55, *p* = 0.29), respectively). The heterogeneity across studies at baseline and at post-treatment 12–14 weeks were over 50% (Figure 4). The results of the ATM were non-significant (slope estimation: −0.15, 95% CI −0.55 to 0.24, *p* = 0.43) (Figure 3B).

### 3.5. Visual Analogue Scale

**Figure 5:**
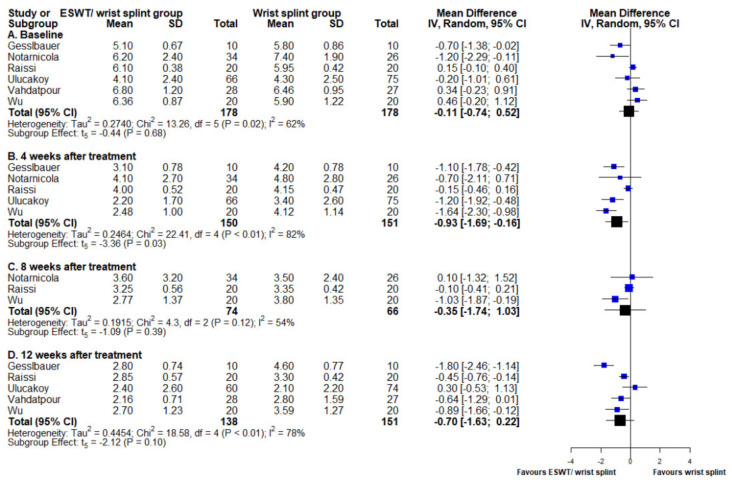
Forest plot of mean difference in VAS between ESWT/night wrist splint and night wrist splint alone groups at (A) baseline, (B) 4 weeks after intervention, (C) 8 weeks after intervention, and (D) 12 weeks after intervention. Mean difference and standard deviation of each enrolled study and the meta-analysis results were presented as blue and black squares at the left column of the figure, respectively.

Six studies included VAS as the outcome [16,17,18,19,20,22]. The ESWT/night wrist splint group showed significant lower VAS scores at 4 weeks of follow-up (MD −0.93, 95% CI −1.69 to −0.16, *p* = 0.03). VAS scores were lower for the ESWT/night wrist splint group at 8 and 12 weeks post-treatment without statistical significance ((MD −0.35, 95% CI −1.74 to 1.03, *p* = 0.39) and (MD −0.70, 95% CI −1.63 to 0.22, *p* = 0.10), respectively). Heterogeneity was high for all of the outcomes (Figure 5). The results of the ATM were also non-significant (slope estimation: −0.03, 95% CI −0.24 to 0.18, *p* = 0.58). (Figure 3C)

### 3.6. Distal Motor Latency of Median Nerve and Sensory Nerve Conduction Velocity

Five studies performed electrodiagnostic evaluations on distal motor latency of the median nerve at baseline and at 12–16 weeks post-treatment [16,17,18,20,22]; five studies tested sensory nerve conduction velocity between the ESWT/night wrist splint group and night wrist splint alone group [15,16,19,20,22]. The follow-up results for distal motor latency of median nerve or sensory nerve conduction velocity were non-significant ((MD 0.01, 95% CI −0.30 to 0.31, *p* = 0.95) and (MD 1.47, 95% CI −1.91 to 4.85, *p* = 0.29), respectively) (Figures S4 and S5, Supplementary Materials).

### 3.7. Adverse Effects

None of the seven studies reported serious side effects in the ESWT/night wrist splint and night wrist splint alone groups.

## 4. Discussion

The results of each included study were heterogenous; Wu et al. and Ke et al. reported superior benefits favoring ESWT/night wrist splint compared to night wrist splint alone after 3 months of treatment [15,19]. Vahdatpour et al. recorded that ESWT/night wrist splint attenuated patient symptoms and functional impairment significantly, with the effect lasting over 6 months [18]. In contrast, Raissi et al., Notarnicola et al., and Gesslbauer et al. concluded that both groups had identical improvements measured by BCTQf and BCTQf at the end of the study [16,17,20]. Ulucaköy et al. found significantly improved pain and functionality in all groups, whereas in the group with ESWT/night wrist splint, a greater improvement of hand function and electrophysiological measures was observed [22]. In this context, a meta-analysis of the efficacy and safety of ESWT/night wrist splint for treating CTS was performed. The present meta-analysis showed that ESWT/night wrist splint yielded only transient improvements at 4 weeks of follow-up assessed by BCTQf, BCTQf, and VAS compared to night wrist splint alone. No significant improvement in the three outcomes (BCTQf, BCTQs, and VAS) was found at other post-treatment time points or using the ATM approach.

Although the mechanism of ESWT on nerve injury is still not well understood, the therapeutic mechanism of ESWT for CTS is believed to induce neovascularization, tissue regeneration, and reduction of inflammation by transferring energy onto the target area via mechanotransduction at appropriate intensity [23]. The brain-derived neurotrophic factor, which plays a central role in neuronal development, maturation, and survival [24], may also be induced following ESWT as a treatment for peripheral nerve injury. ESWT may also assist muscle regeneration from weakening, especially for the thenar eminence, since brain-derived neurotrophic factor can enhance the expression of vascular endothelial growth factor to induce neovascularization [25,26]. However, significant improvements were only observed at 4 weeks after ESWT/night wrist splint treatment, indicating a transient therapeutic effect. An in vivo research study reported that the expression levels of brain-derived neurotrophic factor could only be kept at a stable level up to 26 days after nerve injury by applying ESWT [27]. This may partly explain the short-term functional recovery and symptom remission of CTS patients observed in the ESWT/night wrist splint group in our meta-analysis.

Other alternative options of conservative treatments have been proposed for treating CTS. Acupuncture is one of the most common conservative therapies in eastern society. However, its effectiveness is still questionable due to a lack of robust evidence [28]. Platelet-rich plasma injection was another promising treatment for CTS. Wu et al. reported that platelet-rich plasma injections significantly reduced pain and daily function impairment compared to a control group [29]. Similar results were observed by Uzun et al., for whom the platelet-rich plasma group showed significant lower BCTQs and BCTQf scores after three months of follow-up in comparison with a corticosteroids injection group [30]. It is hard to determine whether the therapeutic efficacy of ESWT is superior to these two conservative treatments since no direct comparison studies have been performed.

Our study was not the first meta-analysis on the effectiveness and safety of ESWT for CTS. Two recent meta-analyses by Kim et al. and Xie et al. reported that ESWT can improve symptoms, functional outcomes, and electrophysiologic parameters in patients with CTS [7,31]. However, the conclusion from these meta-analysis studies might be limited by incorporating discrepant control group datasets combining sham, night wrist splint alone, and local corticosteroid injection as comparison. By appropriate categorization and restricting selection of the included trials, as well individual analysis at different time points, we are convinced that our meta-analysis provides more comprehensive and reliable results.

## 5. Limitations

We acknowledge that there are several limitations in our study. First, the number of included studies and patients were small, potentially leading to high variability and undermining internal and external validity. Secondly, most of the included studies had follow-up period ranging from four weeks to 14 weeks and the long-term clinical efficacy of ESWT remained unclear. Nevertheless, no substantial therapeutic benefit was observed at 8–10 and 12–14 weeks post-treatment; moreover, the ATM approach also revealed no significant improvement trend following ESWT over time, indicating that the benefits brought by ESWT was only temporary. Last but not the least, although only RCTs and well-controlled intervention studies were considered for inclusion, potential sources of bias still exist in these trials, which included inadequate methods to conceal random allocation as well as lack of blinding.

## 6. Conclusions

This meta-analysis analyzed all eligible studies and demonstrated that ESWT usage with concurrent with night wrist splint, compared with night wrist splint alone, was a safe but ineffective treatment for facilitating functional recovery or symptoms remission in CTS patients. Future studies should focus on other conservative treatments that may have longer therapeutic duration in CTS patients.

## Figures and Tables

**Table 1:** Characteristics of the included studies.

Study	Inclusion Criteria	Patient Number, *n*, (Male, %)	Age, Years, Mean ± SD	Lesion Site, *n*	Symptom Duration, Months, Mean ± SD	Interventions
Gesslbauer [Austria, 2020]	a. Electrophysiology study confirmed mild to moderate CTS	T: 10 (2) C:10 (4)	T: 55.8 ± 4.66 C: 54 ± 17.4	NA	T: 29 ± 32.89 C: 33.6 ± 44.26	T: One session/week of fESWT for 3 weeks, 500 shocks, 0.05 mJ/mm^2^ pressure + night wrist splint C: Sham ESWT + night wrist splint
Ke [Taiwan, 2016]	a. Disease duration > 3 months b. Tinel sign or Phalen test positive c. Electrophysiology study confirmed mild to moderate CTS	T: 29 (20.7) C: 30 (16.7)	T: 55.45 ± 1.38 C: 58.13 ± 1.13	T: R15, L14 C: R16, L14	T: 35.34 ± 7.45 C: 34.37 ± 5.42	T: One session of rESWT for 2000 shocks, 4 bar pressure + night wrist splint C: Sham rESWT + night wrist splint during study period
Notarnicola [Italy, 2015]	a. Tinel sign and compression test positive b. Electrophysiology study confirmed CTS	T: 34 C: 26	T: 57.1 ± 9.5 C: 60.2 ± 6.6	Intergroup difference, *p* > 0.05	NA	T: One session/week of ESWT for 3 weeks, 1600 shocks at 0.03 mJ/mm^2^ pressure + wrist splint C: Diet supplementary composed mainly of ALA, GLA, and Echinacea + wrist splint for 10 weeks
Raissi [Iran, 2016]	a. VAS ≥ 4 b. Disease duration > 1 month c. Tinel sign or Phalen test positived. Electrophysiology study confirmed mild to moderate CTS	T: 20 (10) C: 20 (5)	T: 46.1 ± 1.95 C: 46.65 ± 2.23	T: R1, L5, B14 C: R3, L6, B11	NA	T: One session/week of rESWT for 3 weeks, 1000 shocks, 1.5 bar pressure + night wrist splint C: Night wrist splint for 12 weeks
Ulucaköy [Turkey, 2020]	a. Electrophysiology study confirmed mild to moderate CTS	T: 47 (17) C: 50 (6)	T: 48.4 ± 10.1 C: 48.5 ± 9.8	NA	T: 33.7 ± 38.1 C: 24.8 ± 31.5	T: One session/week of rESWT for 3 weeks, 1000 shocks at 0.05 mJ/mm^2^ pressure + night wrist splint C: Night wrist splint for 12 weeks
Vahdatpour [Iran, 2016]	a. Tinel sign and compression test positive b. Electrophysiology study confirmed moderate CTS	Male: 9 Female: 51 (Intergroup difference, *p* > 0.05)	T: 51.5 ± 8.5 C: 49 ± 7.3	NA	T: 3.5 ± 0.35 C: 3.72 ± 0.5	T: One session/week of ESWT for 4 weeks with 800, 900,1000, and 1100 shocks, 0.05, 0.07, 0.1, and 0.15 bar pressure + night wrist splint C: Sham ESWT + night wrist splint for 3 months
Wu [Taiwan, 2016]	a. Tinel sign or Phalen test positive b. Numbness in at least two first, second, or third digits c. Electrophysiology study confirmed CTS	T: 20 (10) C: 20 (15)	T: 54.7 ± 7.96 C: 57.8 ± 6.51	T: R9, L11 C: R11, L9	T: 34.1 ± 33.11 C: 36.1 ± 30.8	T: One session/week of rESWT for 3 weeks, 2000 shocks, 4 bar pressure + night wrist splint C: Sham rESWT + night wrist splint during study period

Abbreviation: ALA, alpha lipoic acid; B, bilateral; C, control group; CTS, carpal tunnel syndrome; ESWT, extracorporeal shock wave therapy; fESWT, focused extracorporeal shock wave therapy; GLA, conjugated linoleic acid; L, left hand; R: right hand; NA, not applicable; rESWT, radial extracorporeal shock wave therapy; T, treatment group.

## Supplementary Materials

The following supporting information can be downloaded at: https://www.mdpi.com/article/10.3390/medicina58050677/s1, Figure S1: PubMed: ((extracorporeal shock wave therapy) OR (shock wave) OR (ESWT)) AND ((carpal tunnel syndrome) OR (Median Neuropathy) OR (Compression Neuropathy) OR (Entrapment Neuropathy)) AND ((splint) OR (conservative treatment)); Figure S2: Embase: (‘extracorporeal shock wave therapy’ OR ‘shock wave’ OR ‘eswt’) AND (‘carpal tunnel syndrome’ OR ‘median neuropathy’ OR ‘compression neuropathy’ OR ‘entrapment neuropathy’) AND (‘splint’ OR ‘conservative treatment’); Figure S3: Cochrane Library: (‘extracorporeal shock wave therapy’ OR ‘shock wave’ OR ‘eswt’) AND (‘carpal tunnel syndrome’ OR ‘median neuropathy’ OR ‘compression neuropathy’ OR ‘entrapment neuropathy’) AND (‘splint’ OR ‘conservative treatment’); Figure S4: Forest plot of mean difference in distal motor latency of median nerve between ESWT/night wrist splint and night wrist splint alone groups at (A) baseline and (B) 12–16 weeks after intervention; Figure S5: Forest plot of mean difference in sensory nerve conduction velocity between ESWT/night wrist splint and night wrist splint alone groups at (A) baseline and (B) 12–24 weeks after intervention; Table S1: Methodology quality assessments by RoB2 of the included studies; PRISMA 2009 checklist.
