# Supplementary material for: Extracorporeal Shock Wave Therapy Provides Limited Therapeutic Effects on Carpal Tunnel Syndrome: A Systematic Review and Meta-Analysis
Source: Medicina (Kaunas). 2022 May 19;58(5):677. doi: 10.3390/medicina58050677 (PMC9144370; doi:10.3390/medicina58050677)
Supplement: Supplementary file 1 [file medicina-58-00677-s001.zip › medicina-1688951-supplementay-Table S1.pdf]

Table S1. Methodology quality assessments by RoB2 of the included studies

Domain 1: Risk of bias arising from the randomization process		
**Signalling questions**	**Comments**	**Response options**
**1.1 Was the allocation sequence random?**	Gesslbauer [2020] 1.1: Y 1.2: PY<br>Karataş [2019] 1.1: N 1.2: N<br>Ke [2016] 1.1: Y 1.2: PY<br>Notarnicola [2015] 1.1: Y 1.2: NI<br>Raissi [2016] 1.1: Y 1.2: PY<br>Ulucaköy [2019] Y 1.2: PY<br>Vahdatpour [2016] 1.1: PY 1.2: NI<br>Wu [2016] 1.1: Y 1.2: PY	Y / PY / PN / N / NI
**1.2 Was the allocation sequence concealed until participants were enrolled and assigned to interventions?**		Y / PY / PN / N / NI
**1.3 Did baseline differences between intervention groups suggest a problem with the randomization process?**	Gesslbauer [2020] 1.3: N<br>Karataş [2019] 1.3: N<br>Ke [2016] 1.3: N	Y / PY / PN / N / NI

	Notarnicola [2015] 1.3: N<br>Raissi [2016] 1.3: N<br>Ulucaköy [2019]: N<br>Vahdatpour [2016] 1.3: N<br>Wu [2016] 1.3: N	
**Risk-of-bias judgement**	Gesslbauer [2020] Low<br>Karataş [2019] High<br>Ke [2016] Low<br>Notarnicola [2015] some concerns<br>Raissi [2016] Low<br>Ulucaköy [2019] Low<br>Vahdatpour [2016] Low<br>Wu [2016] Low	Low / High / Some concerns
Optional: What is the predicted direction of bias arising from the randomization process?		NA / Favours experimental / Favours comparator / Towards null /Away from null / Unpredictable
**Domain 2: Risk of bias due to deviations from the intended interventions (*effect of assignment to intervention*)**		
**Signalling questions**	**Comments**	**Response options**
**2.1. Were participants aware of their assigned intervention during the trial?**	Gesslbauer [2020] 2.1: NI 2.2: Y	Y / PY / PN / N / NI

**2.2. Were carers and people delivering the interventions aware of participants' assigned intervention during the trial?**	Karataş [2019] 2.1: NI 2.2: Y<br>Ke [2016] 2.1: PN 2.2: Y<br>Notarnicola [2015] 2.1: NI 2.2: Y<br>Raissi [2016] 2.1: NI 2.2: Y<br>Ulucaköy [2019] 2.1: NI 2.2: Y<br>Vahdatpour [2016] 2.1: NI 2.2: Y<br>Wu [2016] 2.1: NI 2.2: Y	Y / PY / PN / N / NI
**2.3. If Y/PY/NI to 2.1 or 2.2: Were there deviations from the intended intervention that arose because of the trial context?**	Gesslbauer [2020] 2.3: N<br>Karataş [2019] 2.3: N<br>Ke [2016] 2.3: N<br>Notarnicola [2015] 2.3: N<br>Raissi [2016] 2.3: N<br>Ulucaköy [2019] 2.3: N<br>Vahdatpour [2016] 2.3: N	NA / Y / PY / PN / N / NI

	Wu [2016] 2.3: N	
**2.4 If Y/PY to 2.3: Were these deviations likely to have affected the outcome?**		NA / Y / PY / PN / N / NI
**2.5. If Y/PY/NI to 2.4: Were these deviations from intended intervention balanced between groups?**		NA / Y / PY / PN / N / NI
**2.6 Was an appropriate analysis used to estimate the effect of assignment to intervention?**	Gesslbauer [2020] 2.6: Y<br>Karataş [2019] 2.6: Y<br>Ke [2016] 2.6: Y<br>Notarnicola [2015] 2.6: Y<br>Raissi [2016] 2.6: Y<br>Ulucaköy [2019] 2.6: Y<br>Vahdatpour [2016] 2.6: Y<br>Wu [2016] 2.6: Y	Y / PY / PN / N / NI
**2.7 If N/PN/NI to 2.6: Was there potential for a substantial impact (on the result) of the failure to analyse participants in the group to which they were randomized?**		NA / Y / PY / PN / N / NI
**Risk-of-bias judgement**	Gesslbauer [2020] Low<br>Karataş [2019] Low<br>Ke [2016] Low<br>Notarnicola [2015] Low<br>Raissi [2016] Low<br>Ulucaköy [2019] Low	Low / High / Some concerns

	Vahdatpour [2016] Low<br>Wu [2016] Low	
Optional: What is the predicted direction of bias due to deviations from intended interventions?		NA / Favours experimental / Favours comparator / Towards null /Away from null / Unpredictable
**Domain 3: Missing outcome data**		
**Signalling questions**	**Comments**	**Response options**
**3.1 Were data for this outcome available for all, or nearly all, participants randomized?**	Gesslbauer [2020] 3.1: Y<br>Karataş [2019] 3.1: PY<br>Ke [2016] 3.1: Y<br>Notarnicola [2015] 3.1: Y<br>Raissi [2016] 3.1: Y<br>Ulucaköy [2019] 3.1: Y<br>Vahdatpour [2016] 3.1: Y<br>Wu [2016] 3.1: Y	Y / PY / PN / N / NI
**3.2 If N/PN/NI to 3.1: Is there evidence that the result was not biased by missing outcome data?**		NA / Y / PY / PN / N
**3.3 If N/PN to 3.2: Could missingness in the outcome depend on its true value?**		NA / Y / PY / PN / N / NI
**3.4 If Y/PY/NI to 3.3: Is it likely that missingness in the outcome depended on its true value?**		NA / Y / PY / PN / N / NI

**Risk-of-bias judgement**	Gesslbauer [2020] Low<br>Karataş [2019] Low<br>Ke [2016] Low<br>Notarnicola [2015] Low<br>Raissi [2016] Low<br>Ulucaköy [2019] Low<br>Vahdatpour [2016] Low<br>Wu [2016] Low	Low / High / Some concerns
Optional: What is the predicted direction of bias due to missing outcome data?		NA / Favours experimental / Favours comparator / Towards null /Away from null / Unpredictable
**Domain 4: Risk of bias in measurement of the outcome**		
**Signalling questions**	**Comments**	**Response options**
**4.1 Was the method of measuring the outcome inappropriate?**	Gesslbauer [2020] 4.1: N<br>Karataş [2019] 4.1: N<br>Ke [2016] 4.1: N<br>Notarnicola [2015] 4.1: N<br>Raissi [2016] 4.1: N<br>Ulucaköy [2019] 4.1: N<br>Vahdatpour [2016] 4.1: N<br>Wu [2016] 4.1: N	Y / PY / PN / N / NI

**4.2 Could measurement or ascertainment of the outcome have differed between intervention groups?**	Gesslbauer [2020] 4.2: N<br>Karataş [2019] 4.2: N<br>Ke [2016] 4.2: N<br>Notarnicola [2015] 4.2: N<br>Raissi [2016] 4.2: N<br>Ulucaköy [2019] 4.2: N<br>Vahdatpour [2016] 4.2: N<br>Wu [2016] 4.2: N	Y / PY / PN / N / NI
**4.3 If N/PN/NI to 4.1 and 4.2: Were outcome assessors aware of the intervention received by study participants?**	Gesslbauer [2020] 4.3: N<br>Karataş [2019] 4.3: NI<br>Ke [2016] 4.3: N<br>Notarnicola [2015] 4.3: NI<br>Raissi [2016] 4.3: N<br>Ulucaköy [2019] 4.3: N	NA / Y / PY / PN / N / NI

	Vahdatpour [2016] 4.3: NI<br>Wu [2016] 4.3: N	
**4.4 If Y/PY/NI to 4.3: Could assessment of the outcome have been influenced by knowledge of intervention received?**	Karataş [2019] 4.4: PY<br>Notarnicola [2015] 4.4: PY<br>Vahdatpour [2016] 4.4: PY<br><br>Karataş [2019] 4.5: PN<br>Notarnicola [2015] 4.5: PN<br>Vahdatpour [2016] 4.5: PN	NA / Y / PY / PN / N / NI
**4.5 If Y/PY/NI to 4.4: Is it likely that assessment of the outcome was influenced by knowledge of intervention received?**		NA / Y / PY / PN / N / NI
**Risk-of-bias judgement**	Gesslbauer [2020] Low<br>Karataş [2019] Some concerns<br>Ke [2016] Low<br>Notarnicola [2015] Some concerns<br>Raissi [2016] Low	Low / High / Some concerns

	Ulucaköy [2019] Low<br>Vahdatpour [2016] Some concerns<br>Wu [2016] Low	
Optional: What is the predicted direction of bias in measurement of the outcome?		NA / Favours experimental / Favours comparator / Towards null /Away from null / Unpredictable
**Domain 5: Risk of bias in selection of the reported result**		
**Signalling questions**	**Comments**	**Response options**
**5.1 Were the data that produced this result analysed in accordance with a pre-specified analysis plan that was finalized before unblinded outcome data were available for analysis?**	Gesslbauer [2020] 5.1: Y<br>Karataş [2019] 5.1: Y<br>Ke [2016] 5.1: Y<br>Notarnicola [2015] 5.1: Y<br>Raissi [2016] 5.1: Y<br>Ulucaköy [2019] 5.1: Y<br>Vahdatpour [2016] 5.1: Y<br>Wu [2016] 5.1: Y	Y / PY / PN / N / NI
**Is the numerical result being assessed likely to have been selected, on the basis of the results, from...**		

**5.2. ... multiple eligible outcome measurements (e.g. scales, definitions, time points) within the outcome domain?**	Gesslbauer [2020] 5.2: N<br>Karataş [2019] 5.2: N<br>Ke [2016] 5.2: N<br>Notarnicola [2015] 5.2: N<br>Raissi [2016] 5.2: N<br>Ulucaköy [2019] 5.2: N<br>Vahdatpour [2016] 5.2: N<br>Wu [2016] 5.2: N	Y / PY / PN / N / NI
**5.3 ... multiple eligible analyses of the data?**	Gesslbauer [2020] 5.3: N<br>Karataş [2019] 5.3: N<br>Ke [2016] 5.3: N<br>Notarnicola [2015] 5.3: N<br>Raissi [2016] 5.3: N<br>Ulucaköy [2019] 5.3: N<br>Vahdatpour [2016] 5.3: N<br>Wu [2016] 5.3: N	Y / PY / PN / N / NI

**Risk-of-bias judgement**	Gesslbauer [2020] Low<br>Karataş [2019] Low<br>Ke [2016] Low<br>Notarnicola [2015] Low<br>Raissi [2016] Low<br>Ulucaköy [2019] Low<br>Vahdatpour [2016] Low<br>Wu [2016] Low	Low / High / Some concerns
Optional: What is the predicted direction of bias due to selection of the reported result?		NA / Favours experimental / Favours comparator / Towards null /Away from null / Unpredictable
**Overall risk of bias**		
Risk-of-bias judgement	Gesslbauer [2020] Low<br>Karataş [2019] High<br>Ke [2016] Low<br>Notarnicola [2015] Some concerns<br>Raissi [2016] Low<br>Ulucaköy [2019] Low<br>Vahdatpour [2016] Some concerns<br>Wu [2016] Low	Low / High / Some concerns

Optional: What is the overall predicted direction of bias for this outcome?		NA / Favours experimental / Favours comparator / Towards null /Away from null / Unpredictable

This work is licensed under a Creative Commons Attribution-NonCommercial-NoDerivatives 4.0 International License.
