# Supplementary material for: Extracorporeal Shock Wave Therapy Provides Limited Therapeutic Effects on Carpal Tunnel Syndrome: A Systematic Review and Meta-Analysis
Source: Medicina (Kaunas). 2022 May 19;58(5):677. doi: 10.3390/medicina58050677 (PMC9144370; doi:10.3390/medicina58050677)
Supplement: Supplementary file 1 [file medicina-58-00677-s001.zip › Supplementary material_Figures S1-S5.pdf]

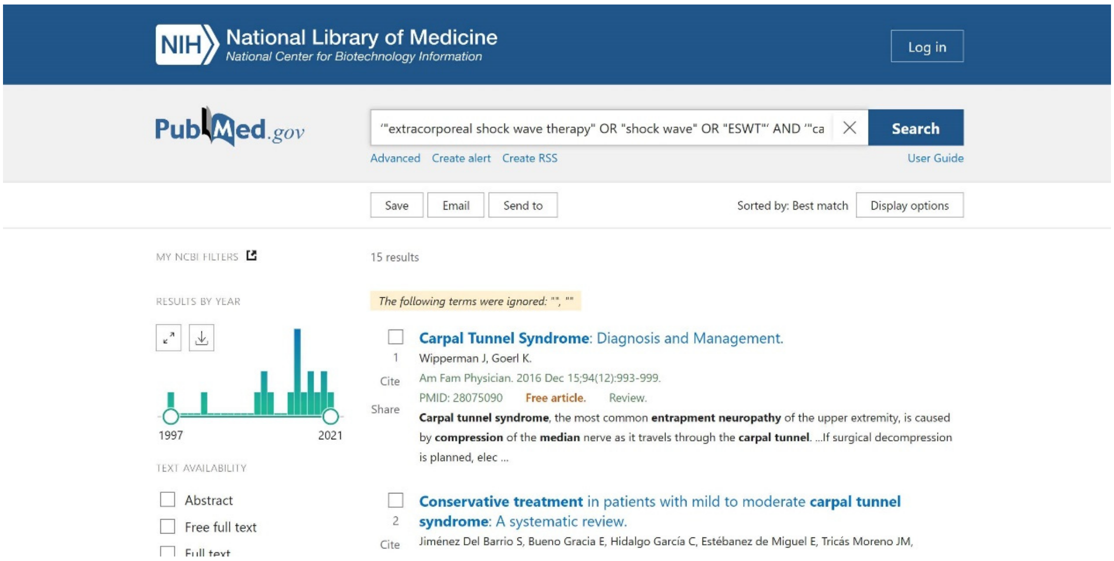

Figure S1. PubMed ((extracorporeal shock wave therapy) OR (shock wave) OR (ESWT)) AND ((carpal tunnel syndrome) OR (Median Neuropathy) OR (Compression Neuropathy) OR (Entrapment Neuropathy)) AND ((splint) OR (conservative treatment)).

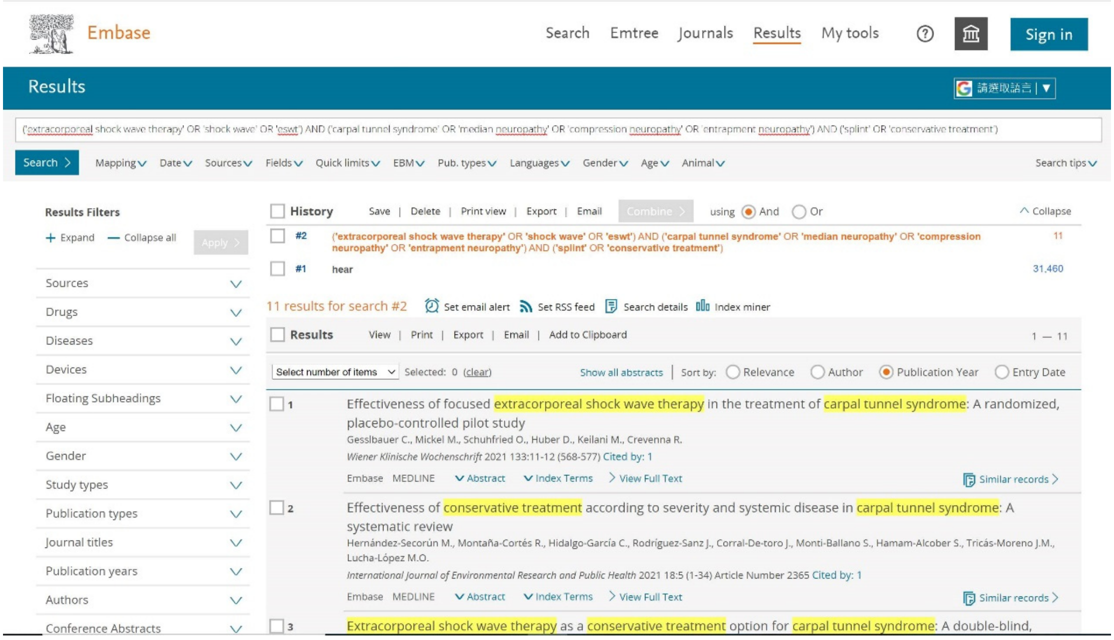

Figure S2. Embase: ('extracorporeal shock wave therapy' OR 'shock wave' OR 'eswt') AND ('carpal tunnel syndrome' OR 'median neuropathy' OR 'compression neuropathy' OR 'entrapment neuropathy') AND ('splint' OR 'conservative treatment').

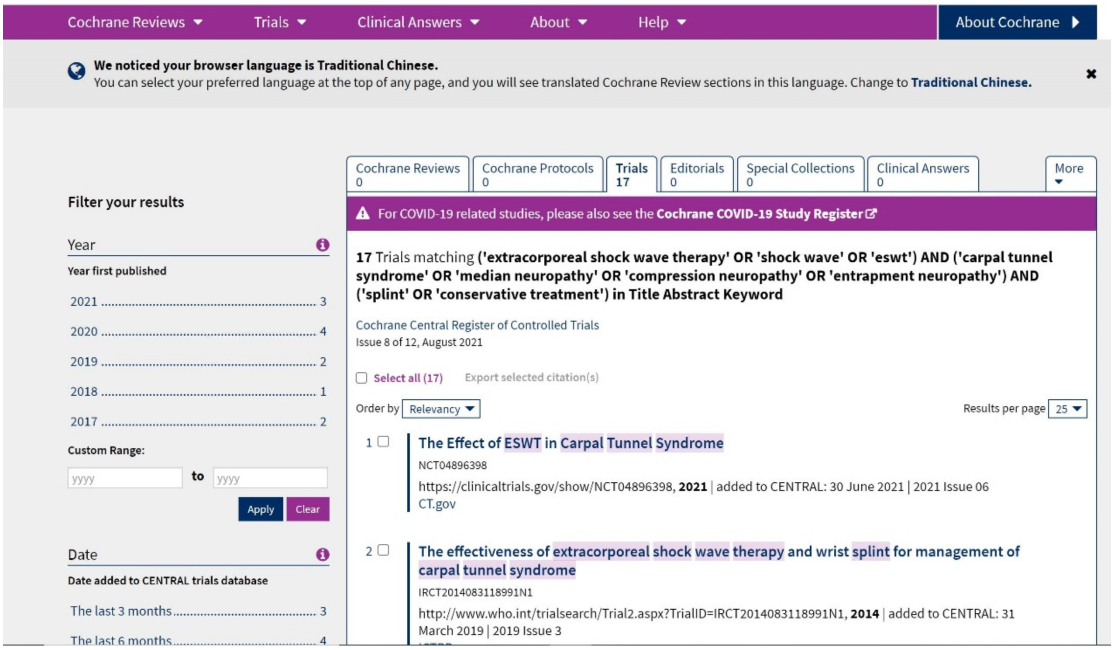

Figure S3. Cochrane Library: ('extracorporeal shock wave therapy' OR 'shock wave' OR 'eswt') AND ('carpal tunnel syndrome' OR 'median neuropathy' OR 'compression neuropathy' OR 'entrapment neuropathy') AND ('splint' OR 'conservative treatment').

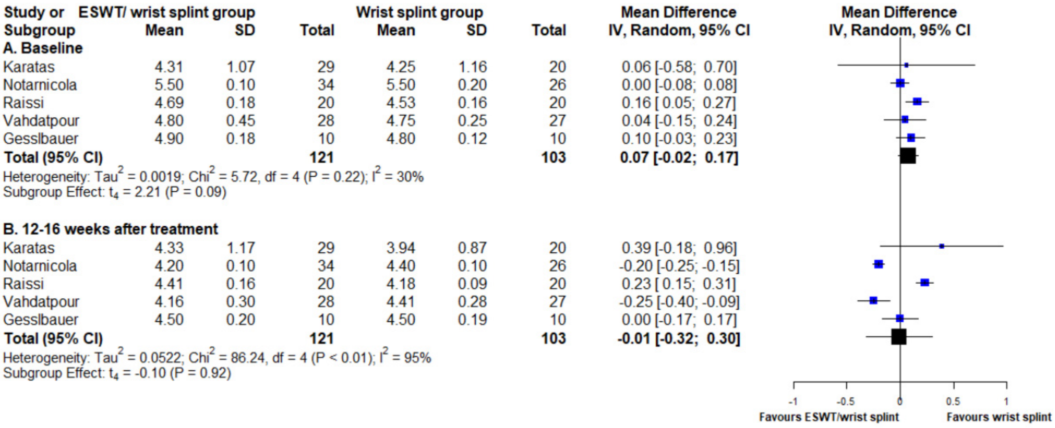

Figure S4. Forest plot of mean difference in distal motor latency of median nerve between ESWT/ night wrist splint and night wrist splint alone groups at (A) baseline and (B) 12-16 weeks after intervention.

Study or Subgroup	ESWT/ wrist splint group Mean	SD	Total	Wrist splint group Mean	SD	Total	Mean Difference IV, Random, 95% CI
**A. Baseline**							
Karatas	38.57	5.41	29	39.90	5.27	20	-1.33 [-4.37; 1.71]
Ke	34.17	1.23	29	32.77	1.33	30	1.40 [0.75; 2.05]
Notarnicola	33.40	0.20	34	33.60	0.40	26	-0.20 [-0.37; -0.03]
Gesslbauer	40.00	1.20	10	38.00	0.46	10	2.00 [1.20; 2.80]
Wu	31.81	5.33	20	30.49	7.28	20	1.32 [-2.63; 5.27]
**Total (95% CI)**			**122**			**106**	**0.81 [-0.73; 2.35]**
Heterogeneity: $Tau^2 = 1.1104$; $Chi^2 = 48.43$, df = 4 (P < 0.01); $I^2 = 92\%$							
Subgroup Effect: $t_4 = 1.46$ (P = 0.22)							
**B. 12-24 weeks after treatment**							
Karatas	40.11	7.21	29	42.77	6.19	20	-2.66 [-6.43; 1.11]
Ke	36.67	1.27	29	34.80	1.39	30	1.87 [1.19; 2.55]
Notarnicola	35.60	0.30	34	35.50	0.20	26	0.10 [-0.03; 0.23]
Gesslbauer	44.00	1.80	10	39.00	0.90	10	5.00 [3.75; 6.25]
Wu	34.92	6.14	20	32.68	8.07	20	2.24 [-2.20; 6.68]
**Total (95% CI)**			**122**			**106**	**1.51 [-1.90; 4.92]**
Heterogeneity: $Tau^2 = 6.2193$; $Chi^2 = 85.52$, df = 4 (P < 0.01); $I^2 = 95\%$							
Subgroup Effect: $t_4 = 1.23$ (P = 0.29)							

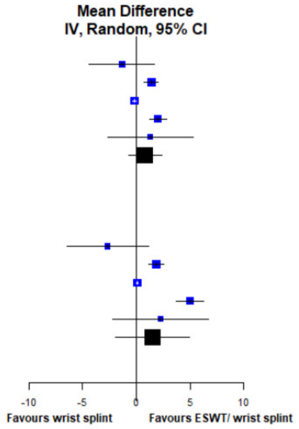

Figure S5. Forest plot of mean difference in sensory nerve conduction velocity between ESWT/ night wrist splint and night wrist splint alone groups at (A) baseline and (B) 12-24 weeks after intervention.
